# Supplementary material for: Emergence of Recombinant Subclade D3/Y in Coxsackievirus A6 Strains in Hand-Foot-and-Mouth Disease (HFMD) Outbreak in India, 2022
Source: Microorganisms. 2024 Feb 28;12(3):490. doi: 10.3390/microorganisms12030490 (PMC10974334; doi:10.3390/microorganisms12030490)
Supplement: Supplementary file 1 [file microorganisms-12-00490-s001.zip › microorganisms-2829561-supplementary.pdf]

Supplementary Table S1: Nucleotide changes and amino acid substitutions identified in VP1 gene (914 bp) of the representative CV-A6 strains with the reference CV-A6 strain (OP896720.1) from Thailand.

NT Position	CV-A6/087		CV-A6/274		CV-A6/051		CV-A6/783	
	**NT changes**	**Amino acid**	**NT changes**	**Amino acid**	**NT changes**	**Amino acid**	**NT changes**	**Amino acid**
2441	-	-	-	-	-	-	T-C	I 573 T
2448	G-A	-	G-A	-	G-A	-	G-A	-
2456	A-C	Q 578 P	A-C	Q 578 P	A-C	Q 578 P	A-C	Q 578 P
2462	A-G	-	A-G	K 580 R	A-G	K 580 R	A-G	K 580 R
2465	T-C	V 581 A	T-C	V 581 A	T-C	V 581 A	T-C	V 581 A
2471	A-G	-	A-G	-	A-G	-	A-G	-
2486	C-T	T 588 I	-	-	-	-	-	-
2531	T-C	L 603 P	T-C	L 603 P	T-C	L 603 P	T-C	L 603 P
2570	C-T	P 616 L	-	-	-	-	-	-
2594	-	-	T-C	-	T-C	-	T-C	-
2630	G-A	-	G-A	-	G-A	-	G-A	-
2657	G-A	R 656 Q	G-A	R 656 Q	G-A	R 656 Q	G-A	R 656 Q
2663	-	-	-	-	-	-	A-G	-
2666	A-G	N 648 S	A-G	N 648 S	A-G	N 648 S	A-G	N 648 S
2678	C-T	P 652 L	-	-	-	-	-	-
2687	G-A	G 655 D	-	-	-	-	-	-
2703	-	-	-	-	-	-	G-A	-
2783	T-C	V 687 A	T-C	V 687 A	T-C	V 687 A	T-C	V 687 A
2787	-	-	-	-	-	-	C-T	-

2813	T-C	L 697 S	T-C	L 697 S	T-C	L 697 S	T-C	L 697 S
2858	G-A	R 712 H	G-A	R 712 H	G-A	R 712 H	G-A	R 712 H
2870	G-A	C 716 Y	G-A	C 716 Y	G-A	C 716 Y	G-A	C 716 Y
2888	A-G	Y 722 C	A-G	Y 722 C	A-G	Y 722 C	A-G	Y 722 C
2897	G-A	G 725 E	G-A	G 725 E	G-A	G 725 E	G-A	G 725 E
2900	T-C	L 726 P	T-C	L 726 P	T-C	L 726 P	T-C	L 726 P
2906	A-G	N 728 S	A-G	N 728 S	-	-	A-G	N 728 S
2957	-	-	-	-	-	-	A-G	Y 745 C
2975	T-C	I 751 T	T-C	I 751 T	T-C	I 751 T	T-C	I 751 T
2978	-	-	A-G	H 752 R	-	-	A-G	H 752 R
3015	A-G	-	-	-	-	-	-	-
3077	T-C	I 785 T	T-C	I 785 T	T-C	I 785 T	T-C	I 785 T
3083	A-G	N 787 S	A-G	N 787 S	A-G	N 787 S	A-G	N 787 S
3158	-	-	-	-	C-T	T 812 M	-	-
3164	T-C	M 814 T	T-C	M 814 T	T-C	M 814 T	T-C	M 814 T
3218	C-T	S 832 F	C-T	S 832 F	C-T	S 832 F	C-T	S 832 F
3221	C-A	A 833 D	C-A	A 833 D	C-A	A 833 D	C-A	A 833 D
3230	T-C	L 836 P	T-C	L 836 P	T-C	L 836 P	T-C	L 836 P
3258	A-G	-	A-G	-	-	-	-	-
3259	-	-	-	-	-	-	G-A	-
3266	T-C	-	T-C	-	T-C	-	T-C	-
3272	C-T	P 850 L	C-T	P 850 L	C-T	P 850 L	C-T	P 850 L
3275	C-T	T 851 I	C-T	T 851 I	C-T	T 851 I	C-T	T 851 I
3278	T-C	L 852 P	T-C	L 852 P	T-C	L 852 P	T-C	L 852 P
3282	A-G	-	A-G	-	A-G	-	A-G	-
3290	T-C	V 856 A	T-C	V 856 A	T-C	V 856 A	T-C	V 856 A
